# 5'-Hydroxymethylcytosine Precedes Loss of CpG Methylation in Enhancers and Genes Undergoing Activation in Cardiomyocyte Maturation

**DOI:** 10.1371/journal.pone.0166575

**Published:** 2016-11-16

**Authors:** David K. Kranzhöfer, Ralf Gilsbach, Björn A. Grüning, Rolf Backofen, Thomas G. Nührenberg, Lutz Hein

**Affiliations:** 1 Institute of Experimental and Clinical Pharmacology and Toxicology, Faculty of Medicine, University of Freiburg, Freiburg, Germany; 2 Bioinformatics Group, Department of Computer Science, University of Freiburg, Freiburg, Germany; 3 University Heart Center Freiburg • Bad Krozingen, Department for Cardiology und Angiology II, Bad Krozingen, Germany; 4 BIOSS Centre for Biological Signaling Studies, University of Freiburg, Freiburg, Germany; Beijing Cancer Hospital, CHINA

## Abstract

**Background:**

Cardiomyocytes undergo major changes in DNA methylation during maturation and transition to a non-proliferative state after birth. 5’-hydroxylation of methylated cytosines (5hmC) is not only involved in DNA loss of CpG methylation but is also thought to be an epigenetic mark with unique distribution and functions. Here, we sought to get insight into the dynamics of 5’-hydroxymethylcytosine in newborn and adult cardiomyocytes.

**Methods:**

Cardiomyocyte nuclei from newborn and adult C57BL/6 mice were purified by flow cytometric sorting. 5hmC-containing DNA was captured by selective chemical labeling, followed by deep sequencing. Sequencing reads of library replicates were mapped independently (n = 3 for newborn, n = 2 for adult mice) and merged for further analysis steps. 5hmC coverage was normalized to read length and the total number of mapped reads (RPKM). MethylC-Seq, ChIP-Seq and RNA-Seq data sets of newborn and adult cardiomyocytes served to elucidate specific features of 5hmC at gene bodies and around low methylated regions (LMRs) representing regulatory genomic regions with enhancer function.

**Results:**

163,544 and 315,220 5hmC peaks were identified in P1 and adult cardiomyocytes, respectively. Of these peaks, 66,641 were common between P1 and adult cardiomyocytes with more than 50% reciprocal overlap. P1 and adult 5hmC peaks were overrepresented in genic features such as exons, introns, 3’- and 5’-untranslated regions (UTRs), promotors and transcription end sites (TES). During cardiomyocyte maturation, 5hmC was found to be enriched at sites of subsequent DNA loss of CpG methylation such as gene bodies of upregulated genes (i.e. *Atp2a2*, *Tnni3*, *Mb*, *Pdk4*). Additionally, centers of postnatally established enhancers were premarked by 5hmC before DNA loss of CpG methylation.

**Conclusions:**

Simultaneous analysis of 5hmC-Seq, MethylC-Seq, RNA-Seq and ChIP-Seq data at two defined time points of cardiomyocyte maturation demonstrates that 5hmC is positively associated with gene expression and decorates sites of subsequent DNA loss of CpG methylation.

## Introduction

Cardiomyocytes represent the contractile cells of the heart. They support cardiac contraction from early embryonic development throughout life. During the prenatal period, the heart grows by proliferation of cardiomyocytes. However, cell division of cardiomyocytes ceases early after birth and the heart continues to grow by hypertrophy, i.e. enlargement of cardiomyocytes [1–3]. In addition to its growth, the heart has to adapt to multiple physiological, biochemical and metabolic transitions during pre- and postnatal life [4, 5]. These adaptations are accompanied by or due to changes in gene expression in cardiomyocytes. Epigenetic mechanisms such as cytosine methylation of DNA and histone modifications accompany and influence cardiac development by regulating transcription [6]. Recent studies have shown that DNA methylation patterns in cardiomyocytes are dynamic during prenatal development and postnatal maturation of the mouse heart [7]. Differential CpG methylation is involved in regulation of developmental stage-specific gene programs both at distal regulatory elements and within gene bodies [7].

DNA methylation at the 5'-cytosine within palindromic CpG sequences (5mC) is involved in fundamental biological processes and is crucial for mammalian development [8]. In 2009, TET1, a Fe^2+^- and α-ketoglutarate-dependent dioxygenase, was discovered to oxidize methylated cytosines to 5’-hydroxymethylcytosine (5hmC) [9]. Subsequent research has detected 5hmC in many tissues and cell types at variable amounts [10–12]. 5hmC can be further oxidized by TET1 and its two isoforms TET2 and TET3 to 5’-formylcytosine (5fC) and 5’-carboxycytosine (5caC) [13, 14]. Removal of 5caC by thymine DNA glycosylase (TDG) and excision of the abasic site by base excision repair (BER) mechanisms may lead to net DNA loss of CpG methylation [13, 15, 16]. Mapping in the endogenous context as well as after TDG deletion revealed that 5fC and 5caC are located at gene regulatory regions such as enhancers and promoters [17, 18]. Despite recent evidence for attenuation of polymerase II transcription elongation by 5caC [19], the functional role of these later 5mC oxidation products still remains to be fully understood. Genome-wide mapping in a variety of tissues and cell types at different developmental time points suggest a role of 5hmC in DNA loss of CpG methylation at specific regulatory elements, namely enhancers [20–24]. Yet, only one report assessed 5hmC at more than one single time point during brain development in the mouse. While demonstrating enrichment of 5hmC at sites acquiring enhancer function, this analysis was not cell-type specific and did not relate to specific genes involved in neuronal development [22]. Besides its role as an intermediate in DNA loss of CpG methylation, 5hmC may also be an epigenetic mark with own regulatory functions. 5hmC was shown to be predominantly stable in various genomic contexts [23, 25–27] and to have its own reader proteins [20, 28–30]. In addition to enhancers [22, 26, 31–36], 5hmC was found to be enriched within gene bodies [22, 26, 32, 34, 35, 37]. In brain tissue [22, 37–39] and in human hepatic tissue [40], 5hmC was shown to be enriched within gene bodies in a transcription level dependent manner.

We hypothesized that 5hmC is dynamically regulated in cardiomyocytes during postnatal maturation. Thus, we used an affinity-based method that is highly specific for capture of 5hmC containing DNA fragments [37] to create cell type-specific genome-wide maps of 5hmC at two stages of postnatal cardiomyocyte maturation. First, cardiomyocytes were purified from newborn mouse hearts on postnatal day 1 (P1). At this stage, myocytes are well differentiated, express mostly fetal gene isoforms and can still proliferate [1, 7]. The second group of cardiomyocytes was derived from mature, adult mouse hearts, which mostly represent terminally differentiated cells with minimal degrees of cell division [1–3, 41]. The results of these 5hmC sequencing experiments were set into context with high-resolution bisulfitomes [7] in order to unveil methylation dynamics both at distal regulatory elements and within genic regions. Our results show that 5hmC is enriched at specific genomic regions that undergo subsequent loss of CpG methylation. On the one hand, gene bodies of genes that become highly expressed in adult cardiomyocytes such as *Atp2a2*, *Tnni3*, *Mb* and *Pdk4* show high levels of 5hmC. On the other hand, 5hmC was enriched at sites of newly established *enhancers* that are in the vicinity of genes which are upregulated during postnatal cardiomyocyte maturation.

## Results

**Fig 1:**
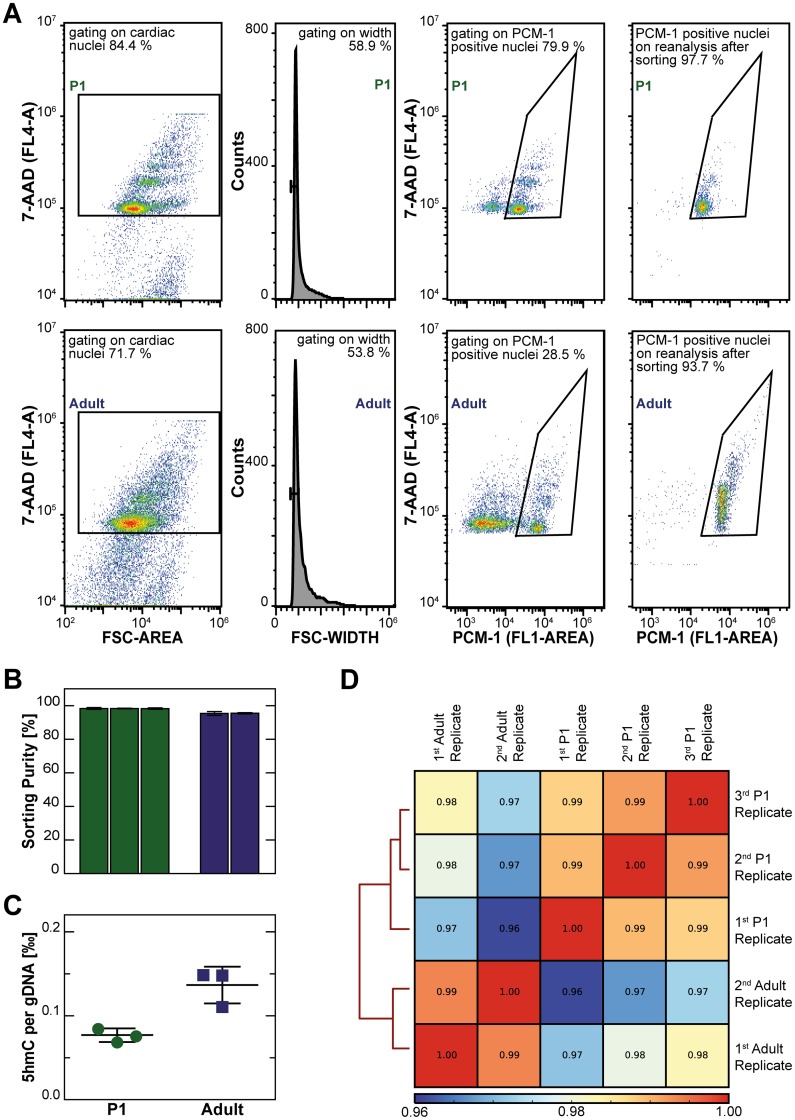
Purification of cardiomyocyte nuclei by flow cytometric cell sorting, global quantification of 5hmC, and sample correlation of 5hmC profiling. (A) Representative flow cytometry plots for one day old (P1, upper panels) and adult (lower panels) mouse hearts. Cardiac nuclei were identified by nuclear staining with 7-AAD and by relative size (FSC-A, first panel), followed by removal of doublets (second panel). Cardiomyocyte nuclei showed strong PCM-1 staining (third panel) and were sorted with high purity as assessed by reanalysis (fourth panel). (B) Percentage of PCM-1 positive nuclei on reanalysis of sorted cardiomyocyte nuclei is given as mean ± SD (%) of three independent sorts per biological replicate. (C) Amount of 5hmC per genomic DNA of three biological replicates of P1 and adult cardiomyocytes is given in weight ‰. (D) The clustered heatmap shows the pair-wise Spearman correlation values of aligned reads mapping to protein coding genes after removal of PCR duplicates.

Cardiomyocyte nuclei from one day old (P1) and from adult 12 week-old mice were identified by an antibody against pericentriolar material 1 protein (PCM-1) [7, 42, 43] and were sorted by flow cytometry (Fig 1A). Cardiomyocyte nuclei were harvested with high purity, reaching 98.3 ± 0.3% for neonatal hearts and 95.4 ± 0.7% for adult hearts (Fig 1B). DNA was isolated from purified cardiomyocyte nuclei and their absolute 5hmC amount determined by a colorimetric antibody-based assay (Fig 1C). Adult cardiomyocytes showed higher hydroxymethylation levels than P1 cardiomyocytes (0.137 ‰ versus 0.077 ‰ of genomic DNA). In order to evaluate the genome-wide distribution of cytosine hydroxymethylation, cardiomyocyte DNA was subjected to labeling and capture of 5hmC by the hydroxymethyl collector method [26, 37, 39] followed by high-throughput sequencing. A total of 55.6 and 59.9 million paired reads uniquely mapped to the mm9 mouse genome for P1 and adult cardiomyocytes, respectively (Table 1). Aligned reads mapping to gene bodies of protein coding genes showed Pearson correlation values of 0.99 between biological replicates of P1 and adult mice, respectively (Fig 1D). All aligned reads showed inter-replicate Pearson correlation values of 0.95 or higher (S1 Fig)

**Table 1:** Sequencing statistics.

Time point	Number of replicate	Number of hearts	Number of reads
P1	1	6	19,367,840
P1	2	6	19,540,112
P1	3	6	16,738,344
Adult	1	3	26,471,520
Adult	2	3	33,404,286

**Fig 2:**
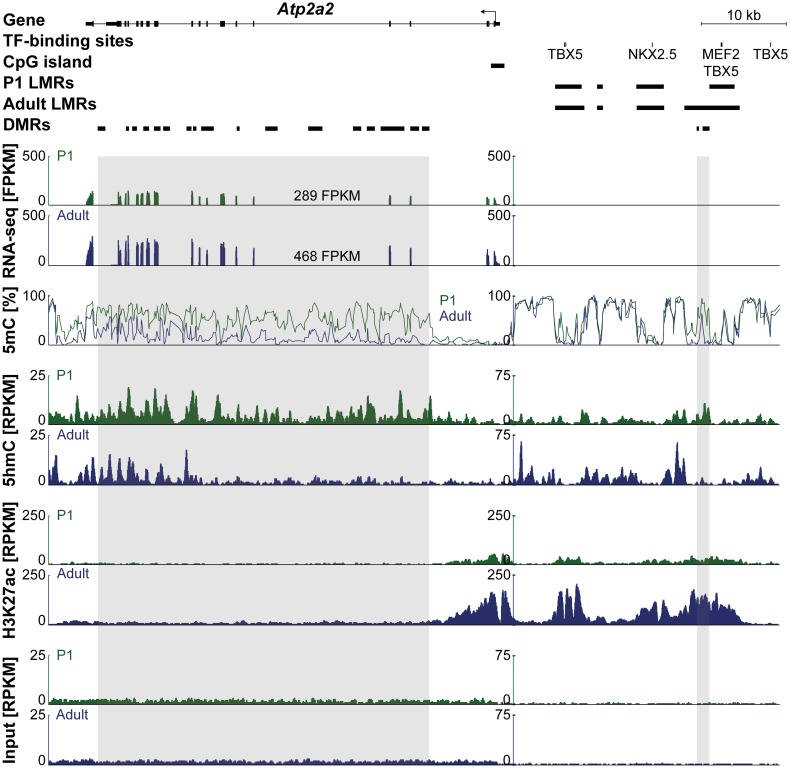
DNA methylation and hydroxymethylation at postnatal day P1 and in adult cardiomyocytes. Genome browser view of the *Atp2a2* gene and an upstream enhancer region with transcription factor (TF)-binding sites and low methylated regions (LMRs). Differentially methylated regions (DMRs) are present in the gene body and at an upstream LMR. RNA-Seq, MethylC-Seq, 5hmC-Seq, H3K27ac ChIP-Seq and input traces (from top to bottom) are shown for P1 (green) and adult (blue) cardiomyocytes.

Inspection of cardiomyocyte genes revealed characteristic patterns of DNA methylation as determined by bisulfite sequencing (5mC) and of hydroxymethylation of DNA (5hmC) (Fig 2). The *Atp2a2* gene encoding for the sarcoendoplasmic reticulum ATPase SERCA showed loss of gene body DNA methylation between neonatal and adult stages which coincided with increased gene expression (Fig 2, traces 1–3). 5hmC was more abundant at the *Atp2a2* gene body of neonatal compared with adult cardiomyocytes (Fig 2, traces 4–5). Furthermore, 5hmC was enriched at differentially methylated regions (DMR) which lost DNA methylation from P1 to adult cardiomyocytes (Fig 2, traces 4–5). Remarkably, 5hmC is depleted in unmethylated regions and is strongly enriched at the 5′-prime border of these regions. Increased expression of *Atp2a2* in adult versus P1 cardiomyocytes was accompanied by higher levels of H3K27 acetylation around the transcription start site and in the 5'-upstream region (Fig 2, traces 6–7).

Dynamic changes in 5hmC patterns were also observed within *Tnni1* and *Tnni3* which represent the fetal and adult isoforms of troponin I1 and I3 genes, respectively (S2A and S2B Fig). Similar to *Atp2a2*, the *Tnni3* gene showed postnatalloss of CpG methylation of the gene body which was pre-marked by higher 5hmC levels in P1 cardiomyocyte nuclei (S2B Fig).

### 5hmC is enriched within genic regions

**Fig 3:**
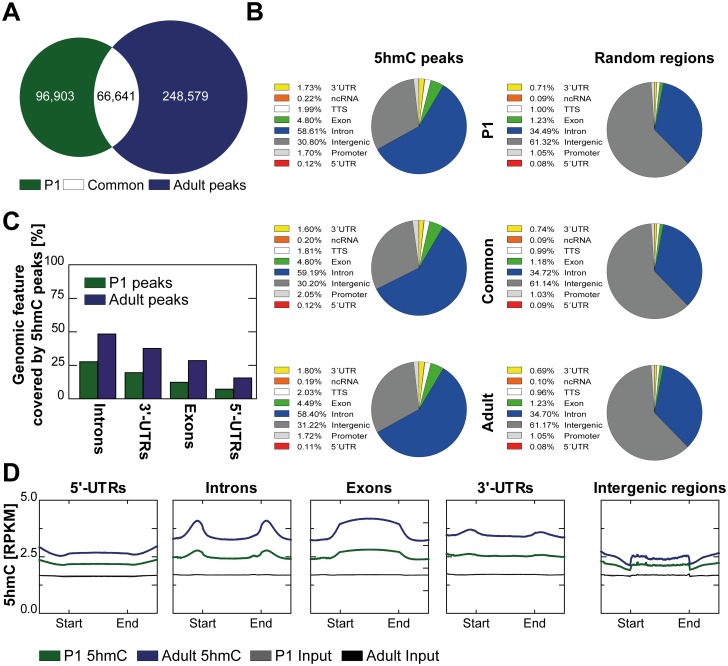
Genomic distribution of 5hmC in P1 and adult cardiomyocytes. (A) Overlap of P1 and adult 5hmC peaks obtained from MACS2 peak calling. Common peaks were defined as P1 and adult peaks sharing at least 50% of their respective length. (B) Annotation of P1, common and adult (from top to bottom) 5hmC peaks obtained from MACS2 peak calling (left panels) and of equally large sets of randomly distributed regions equal in size (right panels) to genomic features is shown. (C) Percentages of genomic features overlapping with 5hmC peaks are given for P1 and adult cardiomyocytes. (D) 5hmC and input coverages in *reads per kilobase per million mapped reads* (RPKM) are depicted for 5’-UTRs, introns, exons, 3’-UTRs and intergenic regions.

163,544 and 315,220 5hmC peaks were identified in P1 and adult cardiomyocytes, respectively (Fig 3A). Of these 5hmC peaks, 66,641 showed a reciprocal overlap of at least 50% and were therefore unambiguously identified as common peaks (Fig 3A). Compared to a random distribution of equally long genomic regions, both P1 and adult only as well as common 5hmC peaks were overrepresented in genic features such as exons, introns, 3’- and 5’-untranslated regions (UTRs), promotors and transcription end sites (TES) (Fig 3B left charts vs. right charts). In contrast, 5hmC peaks were less abundant in intergenic regions as compared with the presence of these regions in the genome (Fig 3B). No change in genome distribution was observed between P1 only, adult only and common peaks (Fig 3B). In P1 and adult mice, introns showed highest overlap with 5hmC peaks, followed by 3’-UTRs, exons and 5’-UTRs (Fig 3C). Average 5hmC coverage was calculated for each genomic feature. Introns and exons, 3’-UTRs and 5’-UTRs showed higher 5hmC coverage than intergenic regions. Exons showed the highest coverage of 5hmC (Fig 3D).

**Fig 4:**
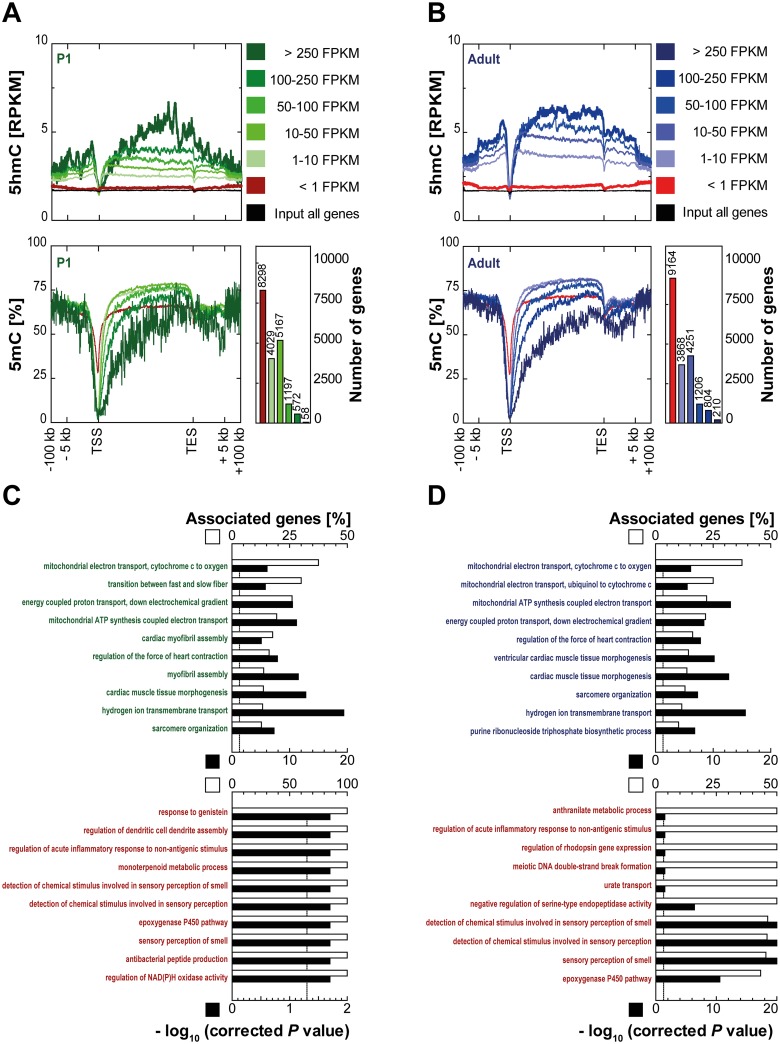
Intragenic 5hmC correlates with transcription. For (A) P1 and (B) adult mice, 5hmC and input coverages (upper panels; RPKM) and methylation profiles obtained from bisulfite sequencing (lower left panels; in %) were plotted for different sets of genes (< 1 FPKM, ≥ 1 and < 10 FPKM, ≥ 10 and < 50 FPKM, ≥ 50 and < 100 FPKM, ≥ 100 and < 250 FPKM, ≥ 250 FPKM) with flanking regions (± 100,000 bp). Input coverages of 5hmC are shown for all protein coding genes. Numbers of genes in each expression category are given (lower right panels). ClueGO was used to identify enriched GO terms (GO term code: biological process) for the genes with very high (upper panels; ≥ 250 FPKM) and low expression (lower panels; < 1 FPKM) in P1 (C) cardiomyocytes and in (D) adult cardiomyocytes. Enriched GO terms were sorted by associated genes per GO term. Dotted lines represent the significance level P < 0.05.

Next, the relation of 5hmC coverage and transcription in cardiomyocytes was analyzed. Transcription start sites (TSS) and gene bodies showed distinct 5hmC patterns both in P1 (Fig 4A) and adult cardiomyocytes (Fig 4B). Irrespective of the gene expression level, transcription start sites were found to be depleted of 5hmC whereas gene bodies were enriched for 5hmC in a transcription level dependent manner (Fig 4A and 4B, upper graphs). Gene body levels of 5hmC increased with higher levels of gene expression in both, P1 and adult cardiomyocytes. The 5hmC content of genes with very low or no expression (<1 FPKM) was similar to that of the input control (Fig 4A and 4B, upper graphs) indicating that presence of genic 5hmC is restricted to expressed genes. Thus, 5hmC patterns showed opposite behavior compared to 5mC levels. Cytosine methylation as determined by bisulfite sequencing was lower at gene bodies of highly expressed cardiomyocyte genes (Fig 4A and 4B, lower graphs) [7].

Next, gene ontology analysis was performed for 50 genes with highest 5hmC levels and gene expression (Fig 4C and 4D, upper panels) and for all genes with an expression level of 0 FPKM (lower panels) of P1 (C) and adult (D) cardiomyocytes. Expectedly, highly expressed genes were linked to energy metabolism and the cardiomyocyte contraction apparatus whereas unexpressed genes showed no enrichment for any cardiomyocyte-related processes. Thus, 5hmC decorates highly expressed genes with cardiomyocyte-specific functions.

### 5hmC precedes loss of CpG methylation of specific genomic loci

Given that both genic and extragenic regions undergo dynamic changes in DNA methylation patterns during postnatal cardiomyocyte development, we hypothesized that 5hmC could indicate genomic loci undergoing subsequent loss of CpG methylation.

#### Gene bodies

**Fig 5:**
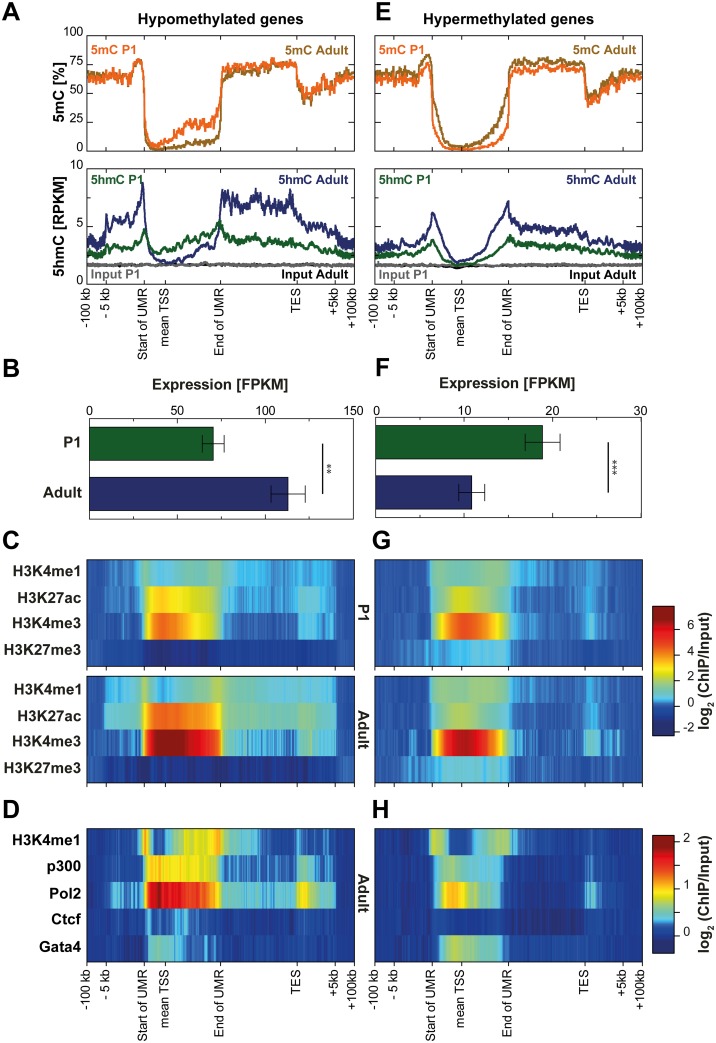
Genic 5hmC dynamics at genes undergoing loss or gain of CpG methylation during postnatal cardiomyocyte maturation. Transcription start sites (TSS) of protein coding genes overlapped by (A) adult unmethylated regions (UMRs) with a mean loss of CpG methylation of at least 5% in adult cardiomyocytes or by (E) P1 UMRs with a mean gain of CpG methylation of at least 5% in adult cardiomyocytes were identified. Methylation profiles obtained from bisulfite sequencing (upper panels; in %) and 5hmC and input coverages (lower panels; RPKM) are shown for the respective regions (± 100,000 bp). Expression levels of genes overlapped by (B) hypomethylated UMRs or by (F) hypermethylated UMRs are given as mean ± s.e.m. Expression differences were analyzed by the Mann-Whitney test (** P < 0.001, *** P < 0.0001). (C, G) Previously published cell type-specific histone modification levels in P1 (upper panels; in log_2_(ChIP/Input)) and adult (lower panels; log_2_(ChIP/Input)) cardiomyocytes as well as (D, H) histone modification/enzyme/transcription factor levels in adult whole heart tissue (log_2_(ChIP/Input)) are shown for genes overlapped by hypomethylated UMRs (left panels) or hypermethylated UMRs (right panels).

Analysis of 5hmC dynamics at genes which are demethylated around their TSS after birth revealed substantial enrichment of 5hmC in P1 compared to adult cardiomyocytes (Fig 5A). This enrichment of 5hmC in P1 cardiomyocytes precisely occurred at sites of later loss of CpG methylation around the TSS. In contrast, the end of the gene body showed much higher enrichment of 5hmC in adult cardiomyocytes compatible with significantly higher expression levels of these genes in adult heart muscle cells (Fig 5B). Increased expression of these genes in adult cardiomyocytes was accompanied by increased active histone modifications (Fig 5C). Of note, occupancy by RNA polymerase II obtained from adult whole heart tissue [44] was particularly high within the demethylated regions (Fig 5D).

Genes with hypermethylation around the TSS after birth (Fig 5E) showed higher 5hmC coverage in adult than in P1 cardiomyocytes. These genes were downregulated (Fig 5F), partly lost the active histone modification H3K27ac (Fig 5G) and showed much less occupancy by RNA polymerase II and P300 [44], compared to demethylated genes (Fig 5H).

Analysis of gene ontology linked genes undergoing DNA loss of CpG methylation to processes important in cardiomyocytes such as mitochondrial energy metabolism and the contraction apparatus (S3 Fig). In contrast, genes which were hypermethylated and repressed postnatally were involved in developmental processes (S3 Fig).

#### Enhancers

**Fig 6:**
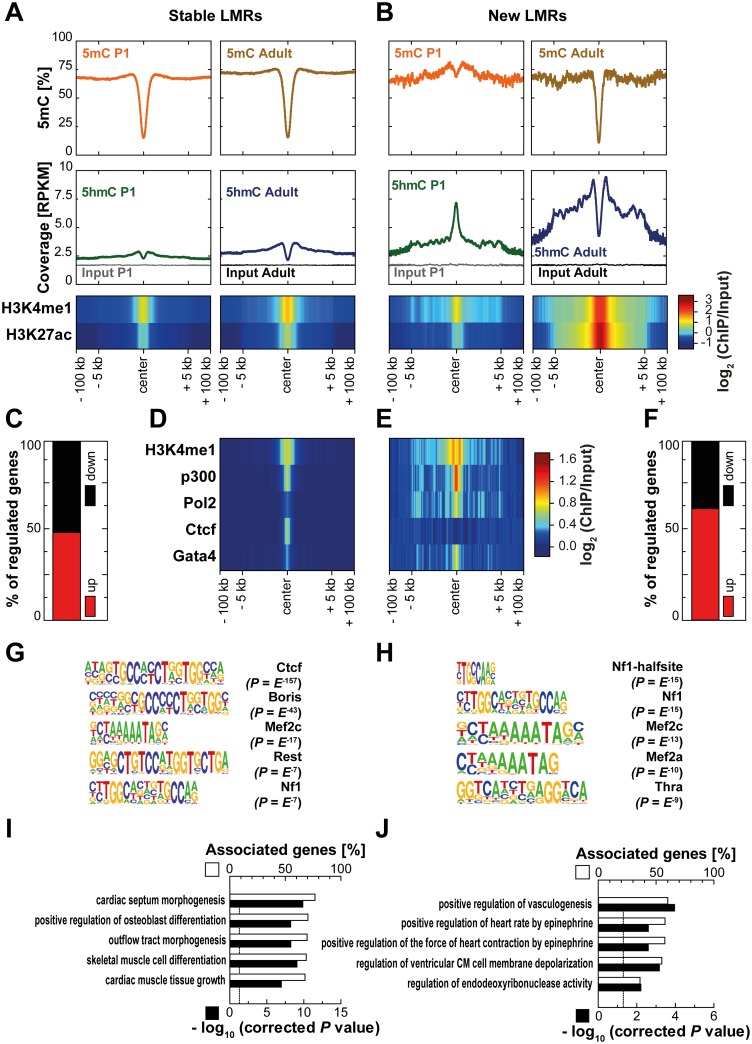
Stable and new low methylated regions (LMRs) show distinct patterns of 5hmC. Methylation profiles obtained from bisulfite sequencing (upper panels; in %) and 5hmC and input coverages (lower panels; RPKM) as well as histone modification levels (log_2_(ChIP/Input)) around (± 100,000 bp) (A) LMRs present at both time points (stable LMRs) and (B) LMRs with a mean loss of CpG methylation of at least 30% in adult cardiomyocytes (new LMRs) are depicted for P1 (left panels) and adult (right panels) cardiomyocytes. Histone modification/enzyme/transcription factor levels in whole heart tissue (log_2_(ChIP/Input)) are shown for (D) stable and for (E) new LMRs. (C, F) Percentage of significantly up- and downregulated genes among all regulated next and second next genes in the vicinity (± 100,000 bp) of (C) stable and (F) new LMRs is shown. The Chi-square test was used to compare both groups (*P* < 0.0001). HOMER was used to identify known motifs of transcription factor-binding sites for (G) stable and for (H) new LMRs. ClueGO was used to identify enriched gene ontology terms (GO term code: biological process) among next and second next genes within ± 100,000 bp near (I) stable and (J) new LMRs. Enriched GO terms were sorted by percentage of associated genes per GO term.

Enhancers are distal regulatory elements positively affecting gene expression through physical interaction with promotor regions [45]. Segmentation of previously published MethylC-Seq data [7, 46] was performed to identify different classes of low methylated regions (LMRs) which have been shown to share features of distal regulatory elements [33]. LMRs both present in P1 and adult cardiomyocytes were termed 'stable LMRs ' (*n* = 10,559). LMRs hypomethylated in adult compared to P1 cardiomyocytes by at least 30% loss of CpG methylation were defined as 'new LMRs' (*n* = 580). Stable LMRs showed a bimodal distribution of 5hmC with depletion at the center and enrichment at the boundaries of the LMR both in P1 and adult cardiomyocytes (Fig 6A). Conversely, new LMRs revealed distinct 5hmC patterns at the two postnatal stages (Fig 6B). In P1 cardiomyocytes, 5hmC showed a strong enrichment at the center even before loss of CpG methylation of the adult LMR occurred. In adult cardiomyocytes, 5hmC was highly enriched at newly established LMRs presenting a bimodal distribution as observed for stable LMRs. (Hydroxy-) Methylation dynamics were paralleled by changes in active histone modifications. Compared to relatively stable levels of H3K4 monomethylation and H3K27 acetylation around stable LMRs (Fig 6A), new LMRs presented a strong gain of these two active marks (Fig 6B). In ChIP-Seq data from adult whole-heart tissue [44, 47], levels of P300, RNA polymerase II and Gata4 were higher around new LMRs than at stable LMRs whereas the latter showed higher enrichment of the transcription factor CTCF (Fig 6D and 6E). Thus, newly established LMRs are mainly enhancers. This is compatible with the observation that nearby genes change transcription. The majority of significantly regulated next and second next genes nearby new LMRs (within ± 100,000 bp) was upregulated between P1 and adult cardiomyocytes (62.4%, *n* = 194, Fig 6F). However, only 49.1% (*n* = 2,385) were significantly higher expressed in the vicinity of stable LMRs (Chi-square test, *P* < 0.0001, Fig 6C). Additionally, upregulation of genes was stronger nearby new than in the vicinity of stable LMRs (*P* < 0.05, data not shown). Next, we applied motif analysis for new and stable LMRs. The CTCF motif was found to be highly enriched within stable LMRs along with BORIS, REST and MEF2C sites whereas new LMRs were enriched for distinct motifs such as THRα (Fig 6G and 6H). Genes in the vicinity of stable LMRs were predominantly associated with cardiogenesis and mesenchymal tissue differentiation. New LMRs were specifically enriched for regulation of cardiomyocyte function (Fig 6I and 6J). Finally, segmentation of the methylomes of three other cell types (mouse embryonic stem cells [33], dermal fibroblasts [48] and NeuN-positive neurons [22] was used to define LMRs present only in adult cardiomyocytes and LMRs shared by all four cell types, termed cardiomyocyte-specific (*n* = 7,766) and constitutive LMRs (*n* = 844), respectively. Both cardiomyocyte-specific and constitutive LMRs showed a bimodal distribution of 5hmC (S4A and S4B Fig) with the enrichment of 5hmC being higher on the boundaries of cardiomyocyte-specific enhancers. In addition, cardiomyocyte-specific LMRs presented higher levels of active histone modifications than constitutive LMRs. Constitutive LMRs showed high CTCF occupancy in adult whole-heart tissue [44] whereas it was virtually absent from cardiomyocyte-specific LMRs (S4D and S4E Fig). GATA4, an important cardiomyocyte transcription factor [47] is enriched at cardiomyocyte-specific LMRs compared to constitutive LMRs (S4D and S4E Fig). Neither up- nor downregulated genes were overrepresented in the vicinity of cardiomyocyte-specific or constitutive LMRs (S4C and S4F Fig). Motif analysis revealed enrichment of the GATA and MEF families of transcription factors for cardiomyocyte-specific LMRs (S4G Fig) whereas constitutive LMRs were enriched for the CTCF motif and the related BORIS motif (S4H Fig). Genes in the vicinity of cardiomyocyte-specific LMRs showed enrichment for biological processes related to cardiomyocyte biology such cardiac septum morphogenesis and outflow tract morphogenesis (S4I Fig) whereas genes near constitutive LMRs showed no enrichment for any heart-related biological processes (S4J Fig). In an additional analysis, stable and new enhancers were identified purely on the histone marks H3K4me1 and H3K27ac and respective dynamics of these marks (S5 Fig). Stable enhancers (*n* = 1,181; S5A Fig) showed, similar to stable LMRs, loss of CpG methylation around the peak center as well as an increase in the bimodal distribution of 5hmC between P1 and adult cardiomyocytes. At new enhancers (*n* = 597; S5B Fig), CpG methylation decreased during cardiomyocyte maturation. 5hmC was enriched at the center of new enhancers in P1 cardiomyocytes, followed by increased enrichment around the center in adult cardiomyocytes.

**Fig 7:**
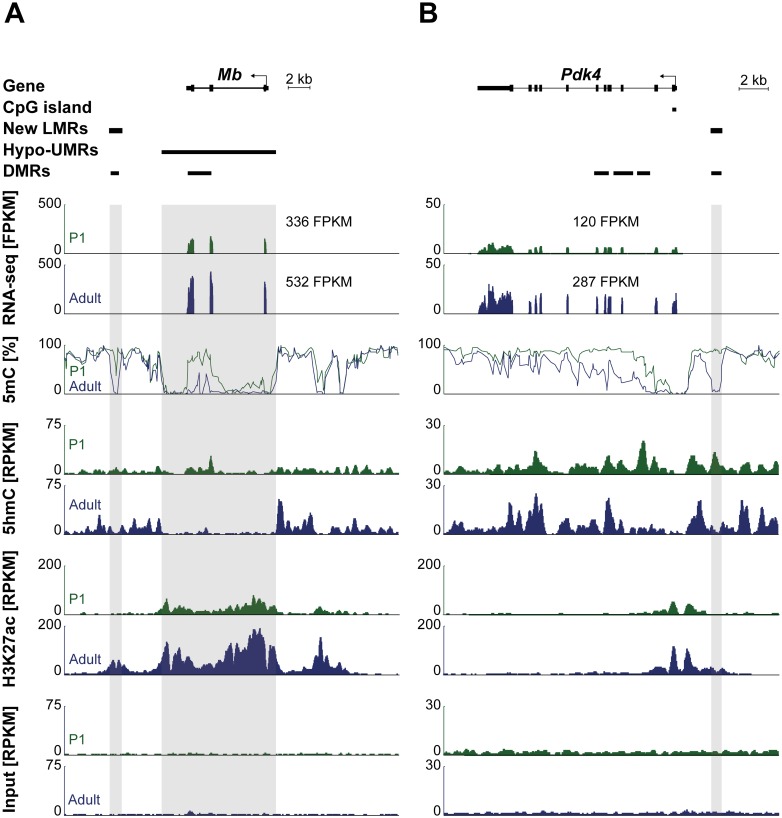
Representative genome browser view of gene body (hydroxy-) methylation and new LMRs. Genome browser view of the *Mb* gene (left) and the *Pdk4* gene (right). Differentially methylated regions (DMRs) are present in the gene body and at a LMR in close relation to the gene. RNA-Seq, MethylC-Seq, 5hmC-Seq, H3K27ac ChIP-Seq and Input traces (from top to bottom) are shown for P1 (green) and adult (blue) cardiomyocytes.

Fig 7 shows example plots of two genes (*Pdk4* and *Mb*) which are significantly upregulated during postnatal cardiomyocyte development in the vicinity of new LMRs decorated by 5hmC in P1 cardiomyocytes.

## Discussion

Genome-wide profiles of 5hmC were generated for proliferating cardiomyocytes from newborn mice and post-mitotic cardiomyocytes from adult mice [49] using selective chemical labeling [37]. High cell type-specificity was achieved by staining with an antibody against a cardiomyocyte-specific protein [7, 42, 43] followed by sorting of nuclei by flow cytometry. Using previously published cardiomyocyte-specific bisulfitomes [7] enabled us to analyze 5hmC in the context of DNA methylation dynamics.

At a global level, we observed an absolute increase in 5hmC amount during postnatal cardiomyocyte maturation. Concurrently, at multiple genomic loci including exons, introns and intergenic regions, we found a relative increase in 5hmC abundance in adult cardiomyocytes. These results are in line with an absolute increase of 5hmC in different neuronal tissues after birth [12, 22, 27, 37]. Interestingly, 5hmC content of a tissue inversely correlates with its proliferation status [25]. Thus, higher 5hmC levels in adult compared to P1 cardiomyocytes might mirror the low proliferation rate of adult cardiomyocytes [49].

Next, we found a correlation of 5hmC content and expression level of a given protein coding gene both in P1 and in adult cardiomyocytes. Previous studies described a similar link between 5hmC and transcription in different tissues and cell types including mouse cerebellum [37], olfactory neurons [38], frontal cortex [22], spermatogenic cells [50] and in human liver [40]. MECP2 was found to bind 5hmC enriched within gene bodies of highly expressed genes in neurons pointing towards a regulatory role of 5hmC in this cell type [29]. Results from TET3 overexpression in mouse olfactory neurons support a positive role for 5hmC in gene expression [38]. However, whether or not transcription might be directly dependent on 5hmC deposition remains elusive. Despite considerable amounts of correlative data, further research is needed to clearly define the potential direct role of 5hmC in gene regulation. This could include identification of (cardiomyocyte-specific) 5hmC reader proteins as well as effects of ablation of TET expression.

Specific genomic loci including gene bodies and nascent enhancers which are demethylated in nuclei after birth exhibited enrichment of 5hmC in P1 compared to adult cardiomyocytes. Loss of CpG methylation in gene bodies near the transcription start site is a feature of cardiomyocyte-specific genes that are strongly upregulated during postnatal maturation [7]. 5hmC enrichment in P1 cardiomyocytes implicates that TET-mediated active loss of CpG methylation takes place at these sites—either through facilitation of passive dilution of 5hmC by DNA replication or by further oxidation of 5hmC and subsequent removal by TDG and BER [51].

Nascent enhancers represent a second genomic element where TET enzymes might be involved in DNA methylation dynamics. Through segmentation of different MethylC-Seq data sets, we identified different classes of LMRs which have been shown to share features of distal regulatory elements [33, 46]. LMRs developing during postnatal cardiomyocyte development are decorated by 5hmC in P1 cardiomyocytes. Subsequent loss of CpG methylation and concomitant increase of active histone marks and upregulation of nearby genes suggest a role of 5hmC in early enhancer activation [20–24, 26]. In contrast, new enhancers in adult cardiomyocytes as well as other classes of distal regulatory elements (stable, cardiomyocyte-specific and constitutive LMRs) at both time points are characterized by depletion of 5hmC in the center of the LMR. When we identified stable and new enhancers based on histone marks, we could confirm the dynamic patterns of CpG methylation and 5hmC observed in the analysis based on LMRs. Yet, loss of CpG methylation, preceding enrichment and subsequent loss of 5hmC at the center of the enhancer were less pronounced. A similar pattern was reported for embryonic stem cells (ESCs) where 5hmC remained enriched at the center of enhancer sites [26, 31–36]. It remains speculative why these different groups of activated enhancers diverge to some extent in their CpG methylation dynamics. Base-resolution analysis of 5hmC in mouse ESCs revealed a local depletion of 5hmC around (± 100 bp) transcription factor binding motifs which is below resolution of affinity-based methods [52]. We found that 5hmC enrichment around LMR centers was very high in new LMRs established during cardiomyocyte maturation whereas stable LMRs showed much lower 5hmC coverage possibly reflecting different TET activities at these two sites. Of note, cardiomyocyte-specific LMRs showed much higher 5hmC enrichment than constitutive LMRs supporting the notion that 5hmC distribution is highly cell type-specific [53].

Aside from loss of CpG methylation in gene bodies and distal regulatory regions, we also noted an accumulation of global 5hmC during cardiomyocyte development as well as 5hmC enrichment in hypermethylated gene bodies. Therefore, 5hmC deposition does not necessarily result in demethylation which is in line with a previous report in developing neurons [39]. Different TET activities at hypomethylated versus hypermethylated regions may control the stability of the 5hmC mark. To determine the functional relevance of TET-mediated 5mC oxidation at cardiomyocyte gene bodies and distal regulatory regions, further studies are required using gene targeting strategies.

In summary, we present highly cell type-specific hydroxymethylomes of proliferating and post-mitotic cardiomyocytes arguing for a role of 5hmC in loss of CpG methylation at gene bodies and nascent enhancers.

## Materials and Methods

### Animal procedures

All animal procedures were performed in accordance with the Guide for the Care and Use of Laboratory Animals published by the National Academy of Sciences 2011 and were permitted by the responsible Committee on the Ethics of Animal Experiments (Regierungspräsidium Freiburg, Germany, permit number: X-12/02S). 12 week old male C57BL/6N mice and one-day old (P1) C57BL/6N mice were sacrificed by cervical dislocation and hearts were removed from the thoracic cavity. Ventricles were isolated, washed in PBS, snap-frozen in liquid nitrogen and stored at -80°C. Tail samples were taken from P1 mice for genotyping and stored at -20°C.

### Genotyping

DNA was extracted from tail samples of P1 mice. The amount of the male-specific *Sry* gene (sex determining region of Chr Y) was assessed by real-time quantitative PCR. Only male mice were used for further experiments. Sense and antisense primer sequences are 5’—GTC CCA CTG CAG AAG GTT GT– 3’ and 5’—CTC ATC GGA GGG CTA AAG TG– 3’, respectively.

### Sorting of cardiomyocyte nuclei

Cardiomyocyte nuclei were sorted as previously described [7, 43] with minor modifications. Briefly, cardiac nuclei were enriched from adult hearts or pairs of P1 hearts using mechanical dissociation followed by sucrose gradient centrifugation. Cardiomyocyte nuclei were stained by an anti-PCM-1 [42] antibody (1:500, HPA023374, Sigma-Aldrich, Taufkirchen, Germany) and an anti-rabbit secondary antibody conjugated to the fluorophore Alexa488 (1:1000, Life Technologies, Karlsruhe, Germany) for 30 minutes at room temperature. All cardiac nuclei were either stained by 7-AAD (1:500, Invitrogen, Karlsruhe, Germany) or Draq-7 (1:100, Cell Signaling Technology, Frankfurt, Germany). Analysis and sorting of cardiomyocyte nuclei was performed using a S3 cell sorter (BioRad, Munich, Germany). A small amount of sorted cardiomyocyte nuclei was reanalyzed in order to determine sorting purity. Sorted cardiomyocyte nuclei were immediately diluted at least 3.5-fold with Buffer RLT Plus (Qiagen, Hilden, Germany) mixed with β-mercaptoethanol (1:100, Merck, Darmstadt, Germany), thoroughly vortexed and stored at -80°C. Flow cytometry experiments were analyzed using the FlowJo software (version 10.0.8r1, Tree Star, Ashland, United States).

### DNA extraction and preparation

Genomic DNA from sorted nuclei was isolated using the AllPrep DNA/RNA Mini Kit (Qiagen, Hilden, Germany) following the protocol supplied by the manufacturer. Concentration of isolated DNA was determined with the Qubit dsDNA HS Assay (Invitrogen, Karlsruhe, Germany). For quantification of 5’-hydroxymethylated DNA, equal amounts of genomic DNA from 6 P1 and 6 adult hearts were pooled to obtain 3 biological replicates, respectively. DNA was concentrated using a Concentrator 5301 (Eppendorf, Wesseling-Berzdorf, Germany) to a final concentration of 24.59 ng/μl. For precipitation of 5’-hydroxymethylated DNA, equal amounts of genomic DNA from 9 pairs of P1 and 6 adult hearts were pooled to obtain 3 P1 and 2 adult biological replicates, each containing 1500 ng of DNA. DNA was sheared in a volume of 100 μl at low intensity for 30 cycles (30 sec ON/ 90 sec OFF) using a Bioruptor (Diagenode, Liège, Belgium). 200 ng of sheared DNA were used for assessment of shearing efficiency by agarose gel electrophoresis (200–500 bp).

### Quantification of 5’-hydroxymethylated DNA

Quantification of 5’-hydroxymethylated DNA was performed using the MethylFlash^™^ Hydroxymethylated DNA Quantification Kit (Epigentek, Farmingdale, United States) following the manufacturer’s instructions with minor modifications. For absolute quantification of hydroxymethylated DNA, a standard curve was generated using different concentrations of a positive control (0.05, 0.1, 0.2, 0.5, 1.0 ng of a polynucleotide containing 20% of hydroxymethylcytosine). Cross-reaction with unmethylated or methylated cytosines was excluded using 1 ng of a polynucleotide containing 20% of cytosines or 20% of methylcytosines, respectively. To evaluate global hydroxymethylation status in cardiomyocytes, 196.7 ng of DNA was used as starting material. Sample as well as control DNA were run in duplicates. Briefly, DNA was bound to assay wells and hydroxymethylated cytosines were detected by a primary capture and a secondary detection antibody. Specific binding to hydroxymethylated DNA was monitored using an enzymatic color reaction. Absorbance was measured at 450 nm using a 2103 EnVision^™^ Multilabel Plate Reader (PerkinElmer, Shelton, United States). For absolute quantification of 5’-hydroxymethylated DNA in cardiomyocytes, a logarithmic standard curve was calculated (*r*^2^ = 0.9591) and 5’-hmC amount determined from OD values as suggested by the manufacturer.

### Precipitation of 5’-hydroxymethylated DNA

Precipitation of 5’-hydroxymethylated DNA was performed using the Hydroxymethyl Collector^™^–Seq Kit (Active Motif, La Hulpe, Belgium) following the manufacturer’s instructions. Briefly, 1000 ng of sheared genomic DNA per biological replicate were used for precipitation. 5’-hydroxymethylcytosine was conjugated to glucose-azide and then biotinylated. Following a purification step, DNA was precipitated using magnetic streptavidin beads. After elution and purification, captured DNA was subjected to library preparation.

### Library preparation and sequencing

Libraries of 5’-hydroxymethylcytosine enriched DNA were prepared using the NEBNext^®^ Ultra Library Prep Kit for Illumina (NEB, Frankfurt, Germany) with minor modifications. Briefly, DNA was end-repaired, ligated to adaptors and amplified by PCR (98°C for 30 seconds, 18 cycles of 98°C for 10 seconds and 65°C for 75 seconds, 65°C for 5 minutes). DNA concentration of PCR amplified libraries was determined using the Qubit dsDNA HS Assay and 5 ng of DNA were subjected to library quality control using High Sensitivity DNA Chips (Agilent, Böblingen, Germany) in combination with the 2100 Bioanalyzer (Agilent, Böblingen, Germany). If appropriate, removal of adaptor and primer dimers was performed with Agencourt AMPure XP Beads (Beckman Coulter, Krefeld, Germany) in a ratio of 0.9 (beads) to 1 (library). Libraries were sequenced on an Illumina HiSeq 2000.

### Bioinformatic analysis

All tools used in this study were integrated into the Galaxy platform. Reads from 5hmC libraries were trimmed using Trim Galore and mapped to the mm9 mouse genome using Bowtie2 [54]. P1 and adult replicates were merged and duplicate reads removed by SAMtools RmDup [55]. RPKM values were obtained using bamCoverage [56]. 5hmC profiles over genomic features were calculated with computeMatrix [56]. PlotCorrelation was used for creating a heatmap of correlation scores (Pearson) among replicates. We used 10.000 bp bins for evaluation of correlation of reads mapping to protein coding genes and 100.000 bp bins for evaluation of correlation of all aligned reads. [56]. MACS2 was used for peak calling [57]. Identified 5hmC peaks were annotated to genomic regions using annotatePeaks.pl of HOMER [58]. The same amount of random regions of equal lengths was created using the BED tool RandomBed [59] and used as background control. Coordinates of different genomic features were downloaded from the UCSC genome browser in order to calculate 5hmC coverage. All sequencing data were deposited at the European Nucleotide Archive (http://www.ebi.ac.uk/ena, study accession number PRJEB14398).

### External Data Sets

Previously published RNA-Seq, ChIP-Seq and MethylC-Seq datasets from P1 and adult cardiomyocytes [7], Chip-Seq data from mouse hearts [44, 47] as well as MethylC-Seq data from embryonic stem cells [33], dermal fibroblasts [48] and NeuN-positive neurons [22] were reanalyzed. RNA-Seq reads were trimmed using Trim Galore and mapped to the mm9 mouse genome using TopHat2 [60]. SAMtools RmDup was used to remove duplicate reads [55]. FPKM expression levels and differential expression testing was performed with Cufflinks and Cuffdiff, respectively [61]. ChIP-Seq and input reads were trimmed with Trim Galore and mapped to the mm9 genome using Bowtie2 [54]. Replicates were merged, duplicates removed by SAMtools RmDup [55] and log_2_ (ChIP/Input) ratios calculated using bamCoverage [56]. Stable and new enhancers were identified based on H3K4me1 and H3K27ac ChIP-Seq data from P1 and adult cardiomyocytes [7], according to the following criteria: New enhancers were defined as adult H3K4me1 and H3K27ac peaks that showed less than 1.3-fold enrichment over input in neonatal but more than 4-fold enrichment over input in adult cardiomyocytes. Stable enhancers were represented by regions that were identified as MACS2 peaks in P1 and adult cardiomyocytes in both histone marks and showed less change in enrichment of the histone marks during cardiomyocyte maturation than the minimal possible change in new enhancers. MethylC-Seq data was mapped with Bismarck [62], followed by further analysis using Methtools (Methtools; https://github.com/bgruening/methtools) as previously described [7].

### Segmentation of MethylC-Seq data

Segmentation of methylomes was performed using the methSeg option of methylKit [46]. For the analysis of gene bodies, UMRs of adult cardiomyocytes were selected if they were overlapping the transcription start site of protein coding genes, but not the transcription end site and displayed a mean methylation change between P1 and adult cardiomyocytes of at least 5%. Stable LMRs were defined as adult cardiomyocyte LMRs overlapping with P1 cardiomyocyte LMRs. New LMRs were defined as adult LMRs with a mean loss of CpG methylation of at least 30% in adult compared to P1 cardiomyocytes. Cardiomyocyte-specific LMRs were defined as LMRs present only in adult cardiomyocytes but not embryonic stem cells, dermal fibroblasts or NeuN-positive neurons whereas constitutive LMRs were present in all four cell types.

### Motif analysis

Motif enrichment analysis for known motifs on the four groups of LMRs (stable, new, cardiomyocyte-specific, constitutive) was performed using findMotifsGenome.pl of HOMER [58], using the ‘-size given’ parameter. Predicted binding sites were taken from a previous publication [7].

### Gene ontology analysis

Gene ontology analysis was performed using the ClueGO software v2.2.5 [63, 64]. Parameters were set as follows: Min GO Level = 6, Max GO Level = 7, GO Fusion = true, Kappa Score Threshold = 0.3, GO_BiologicalProcess-GOA_09.02.2016_16h18, statistical test used = Enrichment/Depletion (Two-sided hypergeometric test), correction method used = Benjamini-Hochberg, number of genes = at least 3, min percentage = at least 5. Significantly enriched GO terms (*P* < 0.05) were sorted by associated genes (%) per GO term.

### Statistical analysis

Most statistical analyses were performed using GraphPad Prism version 4.01 (GraphPad Software, San Diego, United States). Two groups were analyzed using the Mann-Whitney test. Distribution of up- and downregulated genes in the vicinity of LMRs were compared using the Chi-square test. P values smaller than 0.05 were considered significant. Significance levels of differential gene expression between P1 and adult cardiomyocytes as measured by RNA-Sequencing were directly taken from the Cuffdiff output [61].

## Supporting Information

S1 Fig Whole genome sample correlation of 5hmC profiling. The clustered heatmap shows the pair-wise Spearman correlation values of all aligned reads after removal of PCR duplicates.
(TIF)

S2 Fig DNA methylation and hydroxymethylation at postnatal day P1 and in adult cardiomyocytes. Genome browser view of the *Tnni1* (A) and *Tnni3* (B) genes. RNA-Seq, MethylC-Seq, 5hmC-Seq, H3K27ac ChIP-Seq and Input traces (from top to bottom) are shown for P1 (green) and adult (blue) cardiomyocytes.
(TIF)

S3 Fig Functional annotation of genes undergoing de- or hypermethylation around transcription start sites during postnatal cardiomyocyte maturation. GO terms obtained by analysis with ClueGO (GO Term code: biological process) for the genes with (A) loss of CpG methylation at their transcription start site and (B) for genes with gain of CpG methylation at their transcription start site. Enriched GO terms were sorted by percentage of associated genes per GO term.
(TIF)

S4 Fig Cardiomyocyte-specific and constitutive low methylated regions (LMRs) show different abundances of 5hmC. Methylation profiles obtained from bisulfite sequencing (upper panels; in % and 5hmC and input coverages (lower panels; RPKM) as well as histone modification levels (log_2_(ChIP/Input)) around (± 100,000 bp) (A) LMRs only present in adult cardiomyocytes (cardiomyocyte-specific) and (B) LMRs shared by adult cardiomyocytes, NeuN-positive neurons, embryonic stem cells and fibroblasts (constitutive) are depicted for P1 (left panels) and adult (right panels) cardiomyocytes. Histone modification/enzyme/transcription factor levels in whole heart tissue (log_2_(ChIP/Input)) are shown for (D) cardiomyocyte-specific and (E) constitutive LMRs. Percentage of significantly up- and downregulated genes among all regulated next and second next genes in the vicinity (± 100,000 bp) of (C) cardiomyocyte-specific and (F) constitutive LMRs is shown. The Chi-square test was used to compare both groups (*n*.*s*.). HOMER was used to identify known motifs of transcription factor-binding sites for (G) cardiomyocyte-specific and for (H) constitutive LMRs. ClueGO was used to identify enriched gene ontology terms (GO term code: biological process) among next and second next genes within ± 100,000 bp near (I) cardiomyocyte-specific and (J) constitutive LMRs. Enriched GO terms were sorted by percentage of associated genes per GO term.
(TIF)

S5 Fig Stable and new enhancers show distinct patterns of 5hmC. Methylation profiles obtained from bisulfite sequencing (upper panels; in %) and 5hmC and input coverages (lower panels; RPKM) as well as histone modification levels (log_2_(ChIP/Input)) around (± 100,000 bp) (A) enhancers defined by presence of stable H3K4me1 and H3K27ac peaks at both time points (stable enhancers) and (B) enhancers defined by presence of an adult H3K4me1 peak with increasing (more than 4-fold) enrichment of H3K4me1 and H3K27ac marks in adult cardiomyocytes (new enhancers) are depicted for P1 (left panels) and adult (right panels) cardiomyocytes. Histone modification/enzyme/transcription factor levels in whole heart tissue (log_2_(ChIP/Input)) are shown for (C) stable and (D) new enhancers. HOMER was used to identify known motifs of transcription factor-binding sites for (E) stable and (F) new enhancers.
(TIF)
