# Supplementary figures and images for: 5'-Hydroxymethylcytosine Precedes Loss of CpG Methylation in Enhancers and Genes Undergoing Activation in Cardiomyocyte Maturation
Source: PLoS One. 2016 Nov 16;11(11):e0166575. doi: 10.1371/journal.pone.0166575 (PMC5112848; doi:10.1371/journal.pone.0166575)

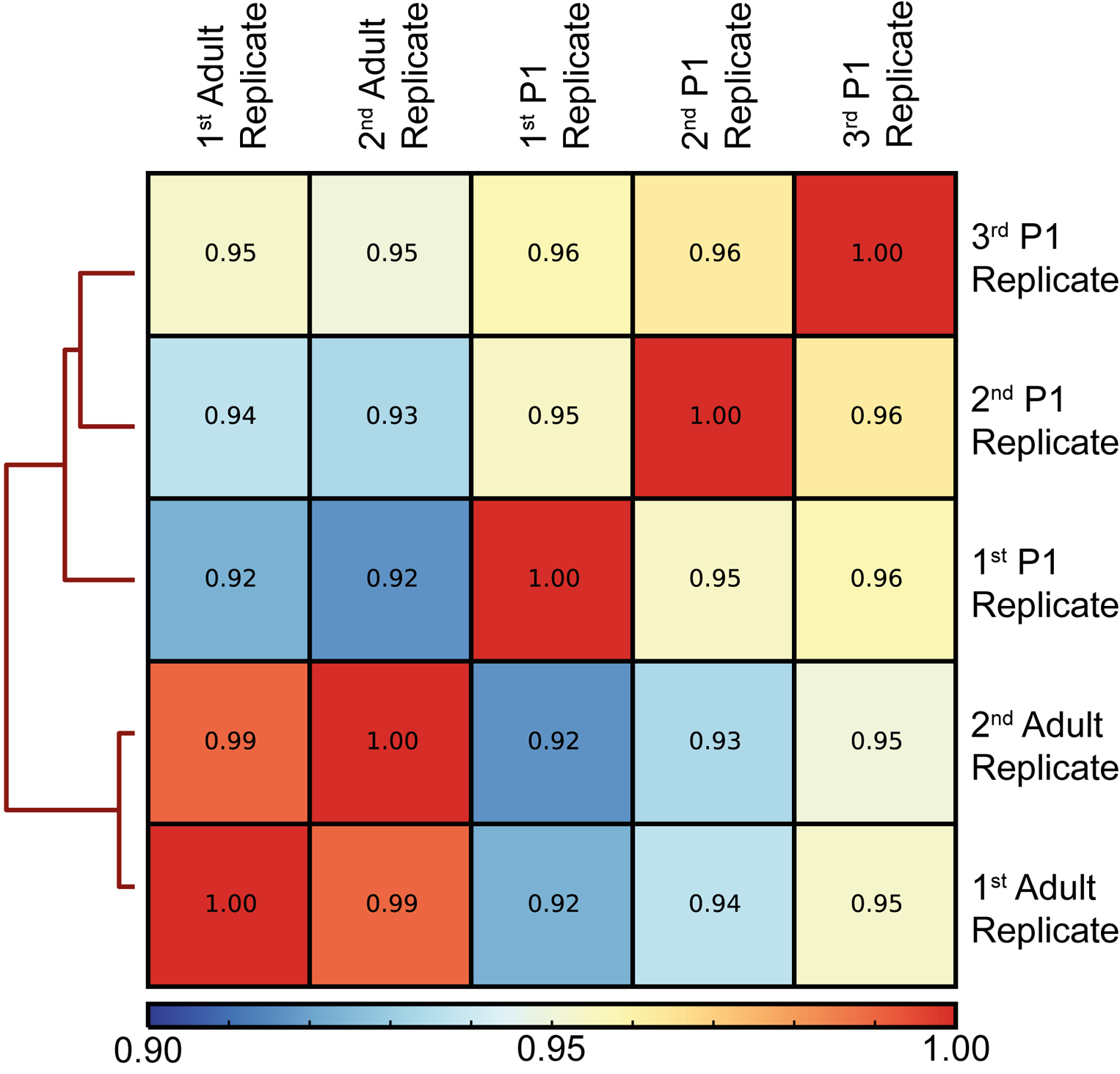

Supplement: S1 Fig — The clustered heatmap shows the pair-wise Spearman correlation values of all aligned reads after removal of PCR duplicates. (TIF) [file pone.0166575.s001.tif]

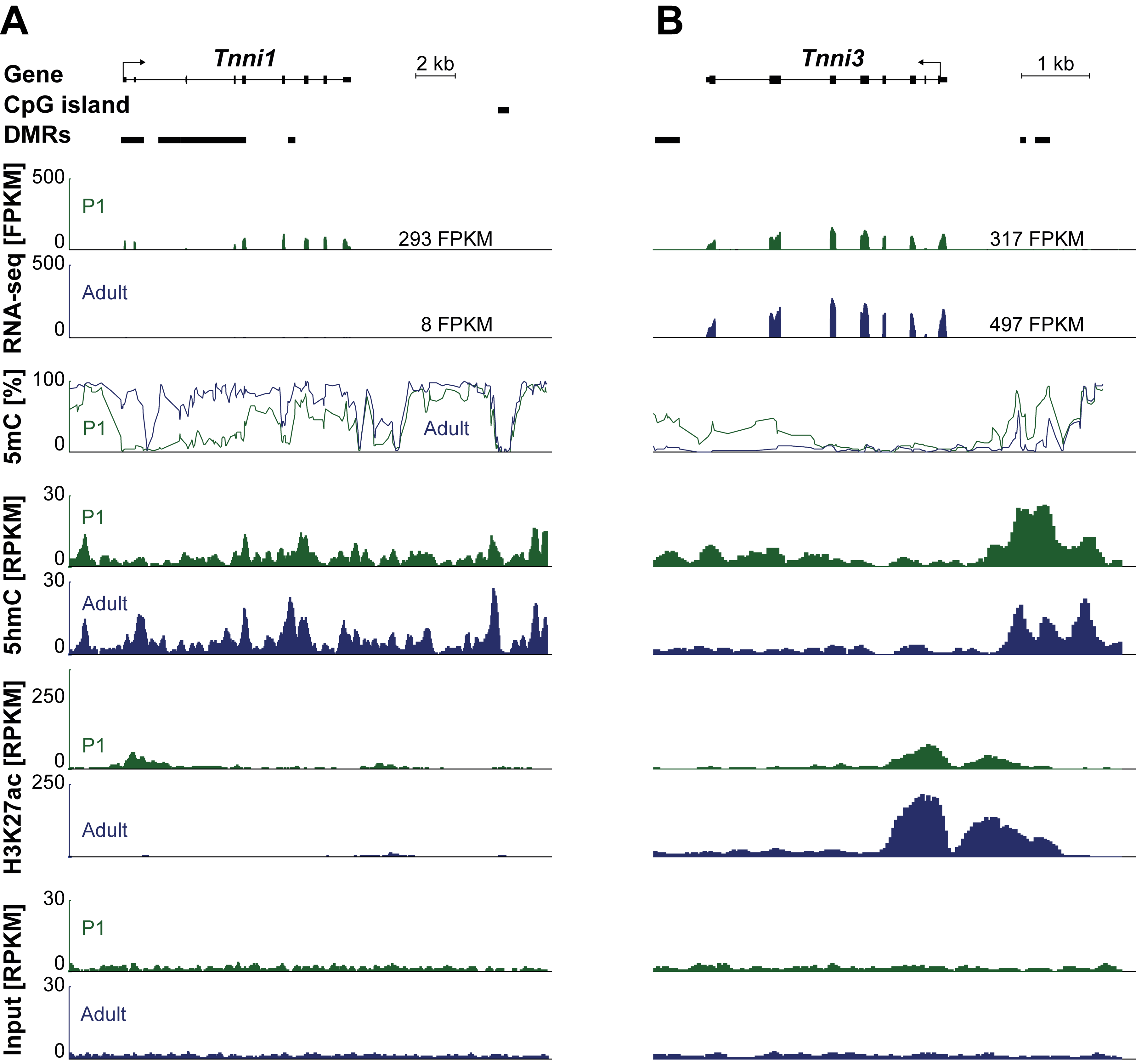

Supplement: S2 Fig — Genome browser view of the Tnni1 (A) and Tnni3 (B) genes. RNA-Seq, MethylC-Seq, 5hmC-Seq, H3K27ac ChIP-Seq and Input traces (from top to bottom) are shown for P1 (green) and adult (blue) cardiomyocytes. (TIF) [file pone.0166575.s002.tif]

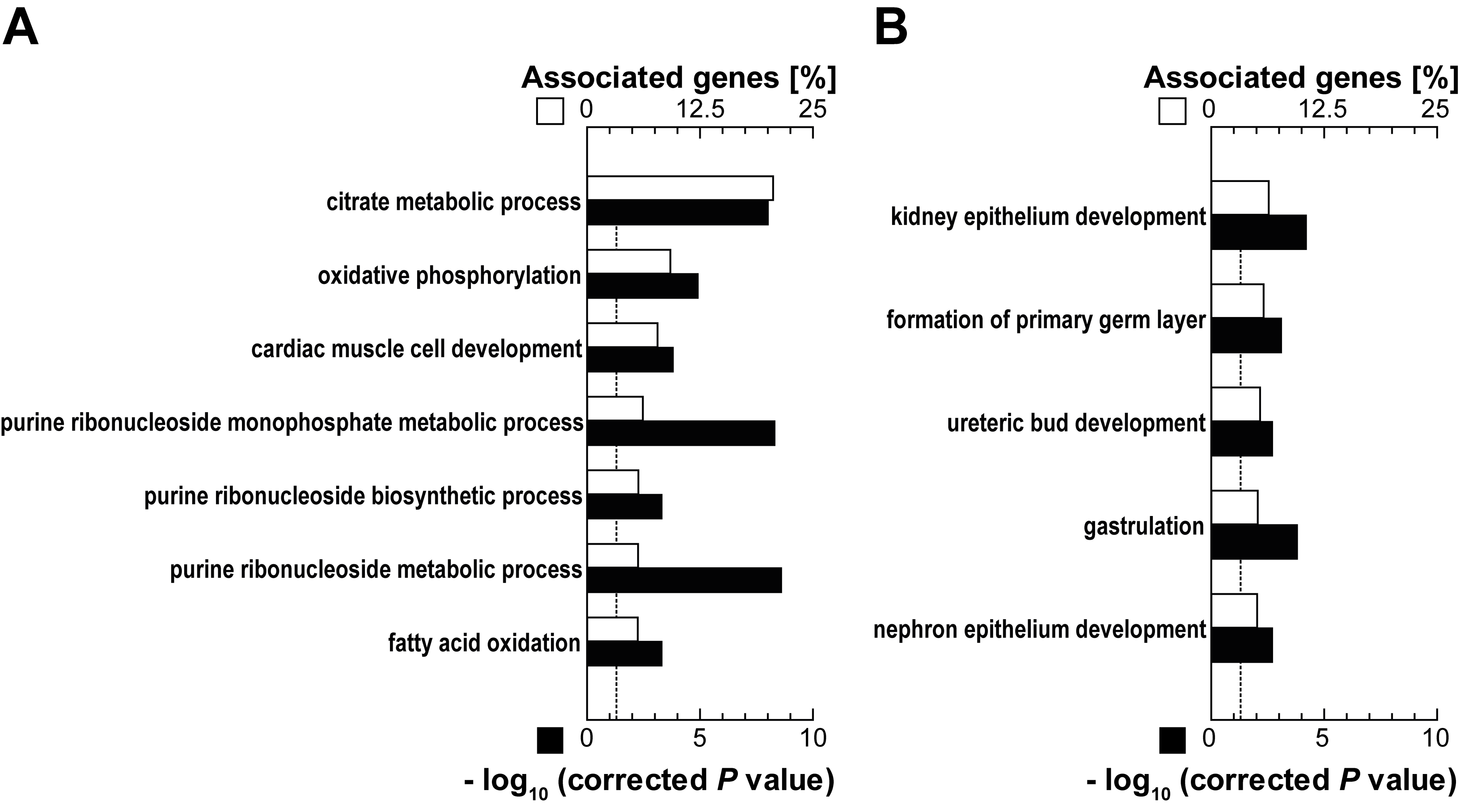

Supplement: S3 Fig — GO terms obtained by analysis with ClueGO (GO Term code: biological process) for the genes with (A) loss of CpG methylation at their transcription start site and (B) for genes with gain of CpG methylation at their transcription start site. Enriched GO terms were sorted by percentage of associated genes per GO term. (TIF) [file pone.0166575.s003.tif]

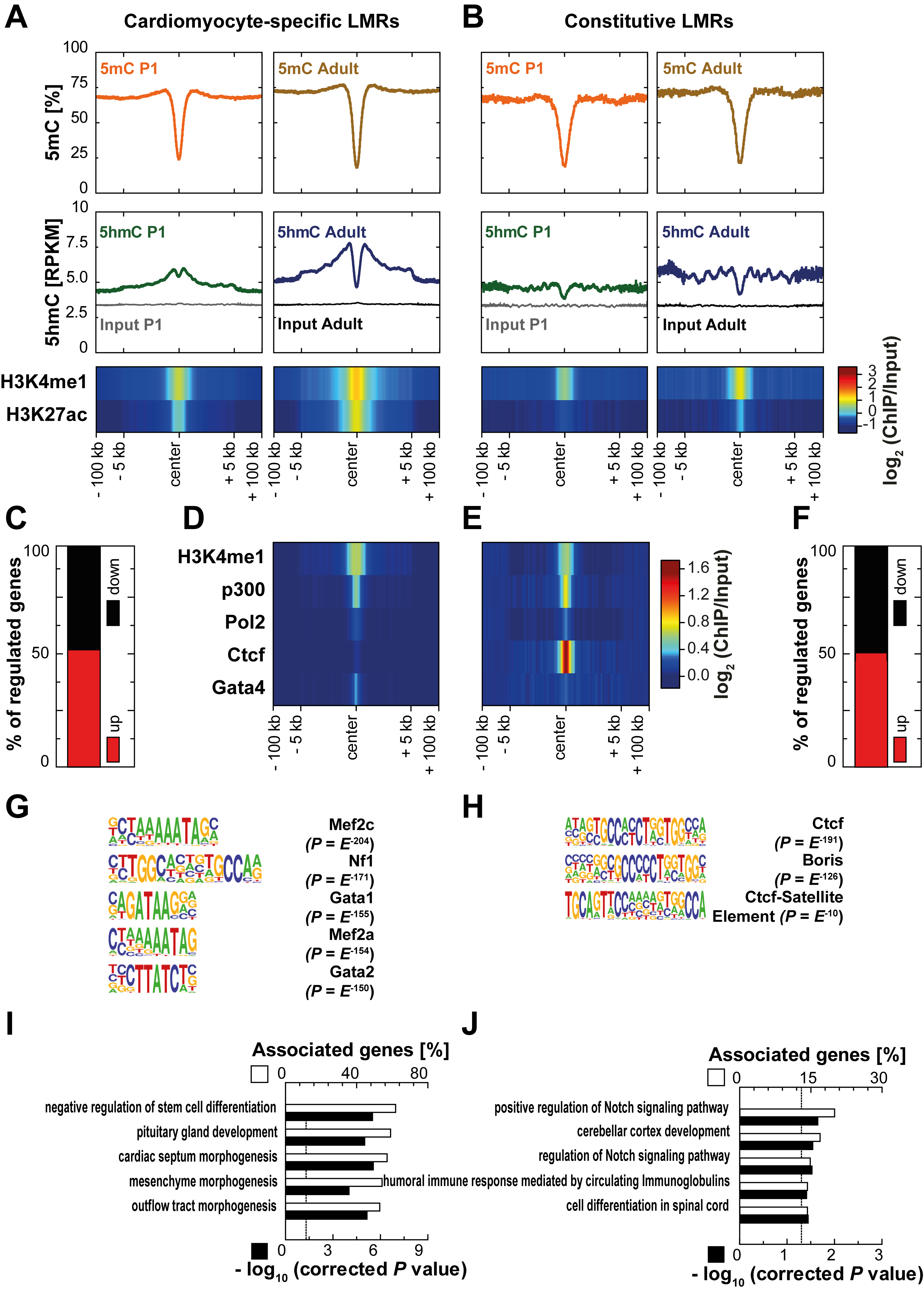

Supplement: S4 Fig — Methylation profiles obtained from bisulfite sequencing (upper panels; in % and 5hmC and input coverages (lower panels; RPKM) as well as histone modification levels (log2(ChIP/Input)) around (± 100,000 bp) (A) LMRs only present in adult cardiomyocytes (cardiomyocyte-specific) and (B) LMRs shared by adult cardiomyocytes, NeuN-positive neurons, embryonic stem cells and fibroblasts (constitutive) are depicted for P1 (left panels) and adult (right panels) cardiomyocytes. Histone modification/enzyme/transcription factor levels in whole heart tissue (log2(ChIP/Input)) are shown for (D) cardiomyocyte-specific and (E) constitutive LMRs. Percentage of significantly up- and downregulated genes among all regulated next and second next genes in the vicinity (± 100,000 bp) of (C) cardiomyocyte-specific and (F) constitutive LMRs is shown. The Chi-square test was used to compare both groups (n.s.). HOMER was used to identify known motifs of transcription factor-binding sites for (G) cardiomyocyte-specific and for (H) constitutive LMRs. ClueGO was used to identify enriched gene ontology terms (GO term code: biological process) among next and second next genes within ± 100,000 bp near (I) cardiomyocyte-specific and (J) constitutive LMRs. Enriched GO terms were sorted by percentage of associated genes per GO term. (TIF) [file pone.0166575.s004.tif]

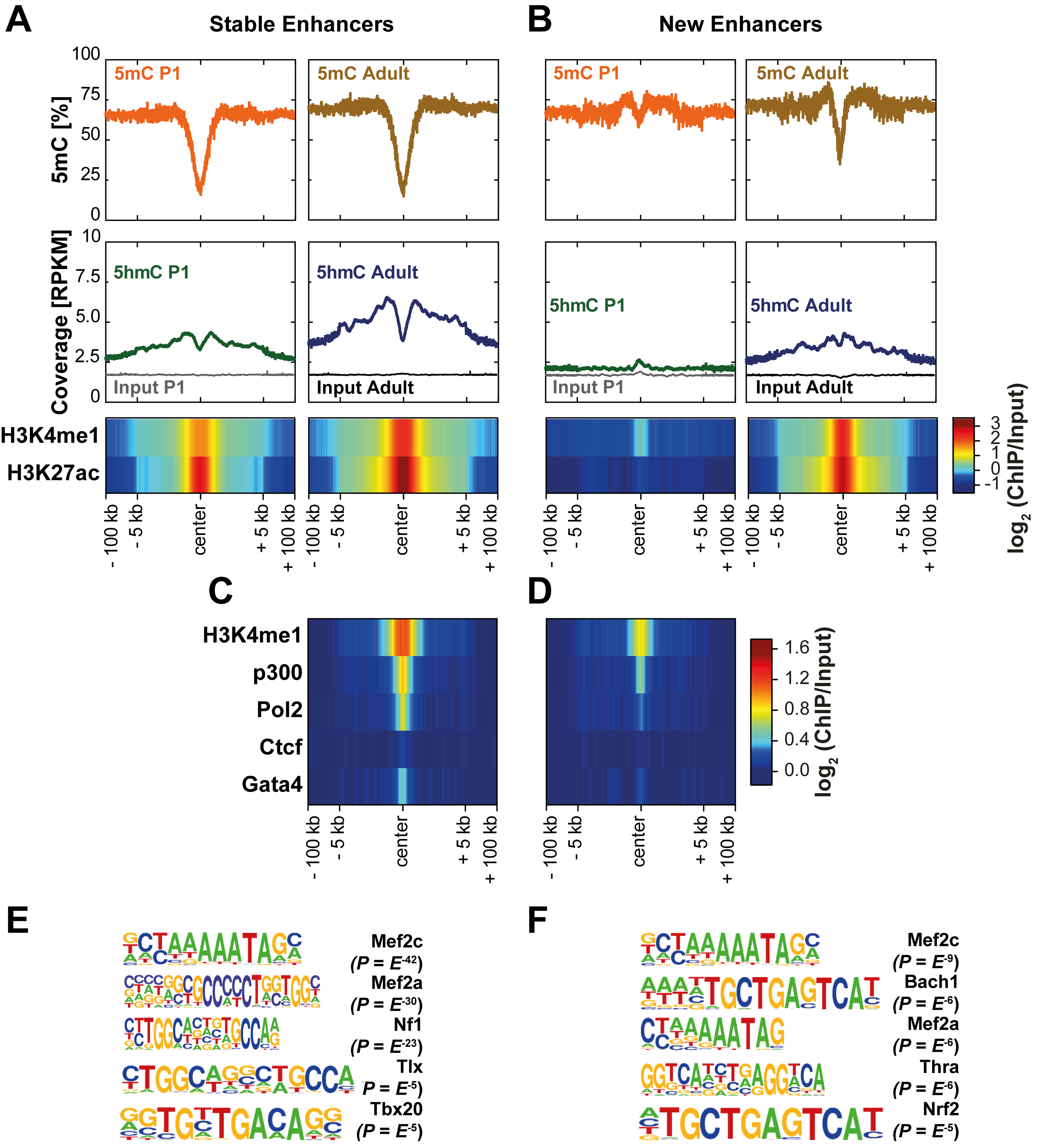

Supplement: S5 Fig — Methylation profiles obtained from bisulfite sequencing (upper panels; in %) and 5hmC and input coverages (lower panels; RPKM) as well as histone modification levels (log2(ChIP/Input)) around (± 100,000 bp) (A) enhancers defined by presence of stable H3K4me1 and H3K27ac peaks at both time points (stable enhancers) and (B) enhancers defined by presence of an adult H3K4me1 peak with increasing (more than 4-fold) enrichment of H3K4me1 and H3K27ac marks in adult cardiomyocytes (new enhancers) are depicted for P1 (left panels) and adult (right panels) cardiomyocytes. Histone modification/enzyme/transcription factor levels in whole heart tissue (log2(ChIP/Input)) are shown for (C) stable and (D) new enhancers. HOMER was used to identify known motifs of transcription factor-binding sites for (E) stable and (F) new enhancers. (TIF) [file pone.0166575.s005.tif]
